# Cognitive Training and Transcranial Direct Current Stimulation for Mild Cognitive Impairment in Parkinson's Disease: A Randomized Controlled Trial

**DOI:** 10.1155/2018/4318475

**Published:** 2018-03-26

**Authors:** Blake J. Lawrence, Natalie Gasson, Andrew R. Johnson, Leon Booth, Andrea M. Loftus

**Affiliations:** ^1^Curtin Neuroscience Laboratory, School of Psychology and Speech Pathology, Curtin University, Bentley, WA 6102, Australia; ^2^ParkC Collaborative Research Group, Curtin University, Bentley, WA 6102, Australia; ^3^Ear Science Institute Australia, Subiaco, WA, Australia; ^4^Ear Sciences Centre, School of Surgery, The University of Western Australia, Crawley, WA, Australia

## Abstract

This study examined whether standard cognitive training, tailored cognitive training, transcranial direct current stimulation (tDCS), standard cognitive training + tDCS, or tailored cognitive training + tDCS improved cognitive function and functional outcomes in participants with PD and mild cognitive impairment (PD-MCI). Forty-two participants with PD-MCI were randomized to one of six groups: (1) standard cognitive training, (2) tailored cognitive training, (3) tDCS, (4) standard cognitive training + tDCS, (5) tailored cognitive training + tDCS, or (6) a control group. Interventions lasted 4 weeks, with cognitive and functional outcomes measured at baseline, post-intervention, and follow-up. The trial was registered with the Australian New Zealand Clinical Trials Registry (ANZCTR: 12614001039673). While controlling for moderator variables, Generalized Linear Mixed Models (GLMMs) showed that when compared to the control group, the intervention groups demonstrated variable statistically significant improvements across executive function, attention/working memory, memory, language, activities of daily living (ADL), and quality of life (QOL; Hedge's *g* range = 0.01 to 1.75). More outcomes improved for the groups that received standard or tailored cognitive training combined with tDCS. Participants with PD-MCI receiving cognitive training (standard or tailored) or tDCS demonstrated significant improvements on cognitive and functional outcomes, and combining these interventions provided greater therapeutic effects.

## 1. Introduction

There is a growing body of research examining mild cognitive impairment in Parkinson's disease (PD-MCI) and the potential of nonpharmacological interventions (e.g., cognitive training and noninvasive brain stimulation) for improving cognitive function in PD and PD-MCI [1].

There are two frequently used methods of computer-based cognitive training: standard or tailored. Standard cognitive training involves cognitive tasks that are not customised to the individual's cognitive deficits, whereas tailored cognitive training is deficit specific. Recent studies report improved cognition following standard and tailored cognitive training in PD. París et al. [2] examined whether standard multimedia and paper/pencil cognitive training improved cognitive functioning, quality of life (QOL), and activities of daily living (ADL) in PD. Compared to the control group, the trained group improved across all cognitive domains except language, but no improvement was found for QOL and ADL [2]. In a randomized controlled trial, Edwards et al. [3] examined whether standard computer-based cognitive training improved speed of processing in PD. There were significant improvements in speed of processing for those with mild/moderate PD [3]. For tailored cognitive training, Naismith et al. [4] examined the effect of two-hour sessions twice a week, which involved psychoeducation and tailored computer-based tasks. Episodic memory and learning retention significantly improved posttraining [4]. Cerasa et al. [5] examined neurofunctional correlates between trained cognitive domains and synaptic plasticity of those domains in PD. Participants completed 12 hours of computer-based cognitive training tailored to their pretraining cognitive impairment(s). Compared to the control group, the training group demonstrated attentional improvements which increased neural resting state (fMRI) activity in the superior parietal and prefrontal dorsolateral cortices [5]. There is increasing evidence supporting standard and tailored cognitive training for cognition in PD, but it remains unclear which modality has greater therapeutic potential [6].

Transcranial direct current stimulation (tDCS) modulates neuronal activity by delivering low-intensity electrical currents to specific cortical regions [7]. Initial studies report improved cognition following tDCS in PD. Boggio et al. [8] demonstrated that 2 mA tDCS over left DLPFC improved working memory in PD, whereas 1 mA and sham tDCS provided no beneficial effects for cognition. Pereira et al. [9] examined whether 20 minutes of counterbalanced 2 mA tDCS over left DLPFC and left temporoparietal cortices immediately improved executive functions. In a randomized controlled trial of tDCS in PD, Doruk et al. [10] compared 2 mA tDCS applied over left (group one) or right (group two) DLPFC with sham stimulation (control group) for executive function. Compared to the control group, significant improvements in the Trail Making Test (Part B) were found for both tDCS groups immediately following the two-week intervention and at one-month follow-up [10]. These studies provide preliminary evidence that tDCS may improve cognitive function in PD, but more standardised clinical trials are required to substantiate these findings.

One recent study [11] combined cognitive training with tDCS simultaneously and reported a trend towards significant improvement in memory, but the lack of a control group limits interpretation of intervention effects. It remains unclear whether combining cognitive training with tDCS provides optimal conditions (stimulation and compensation) to elicit neuronal plasticity and improve cognition in PD and PD-MCI. The present study examined whether standard cognitive training, tailored cognitive training, tDCS, standard cognitive training + tDCS, and tailored cognitive training + tDCS improved cognitive function and practical outcomes in PD-MCI.

## 2. Methods

### 2.1. Study Design

This study was a parallel, randomized controlled trial conducted in accordance with CONSORT requirements (see Supplementary Table S1) [12]. Participants were randomized to one of six groups (5 intervention and 1 control) by a computer generated list using block randomization at a ratio of 1 : 1. Blinding is difficult to achieve in nonpharmacological trials, and so participants and researchers were not blinded to the interventions.

Participants in the standard or tailored cognitive training groups completed computer-based training for 45 minutes, 3 times per week for 4 weeks. Cognitive training was completed using the website version of Smartbrain Pro™ (http://www.smartbrain.net) in participants' homes. Participants in a tDCS group completed 20 minutes of stimulation, once a week for 4 weeks. Each session of tDCS was completed at Curtin University. All participants completed the same neuropsychological tests at baseline (week 0), post-intervention (week 5), and follow-up (week 12).

Curtin University's Ethics Committee provided approval (approval number: HR 189/2014), and this study was registered with the Australian New Zealand Clinical Trials Registry (ANZCTR: 12614001039673). All participants provided informed consent, and participation was completed during participants' “ON” stage of medication.

### 2.2. Study Population

**Figure 1:**
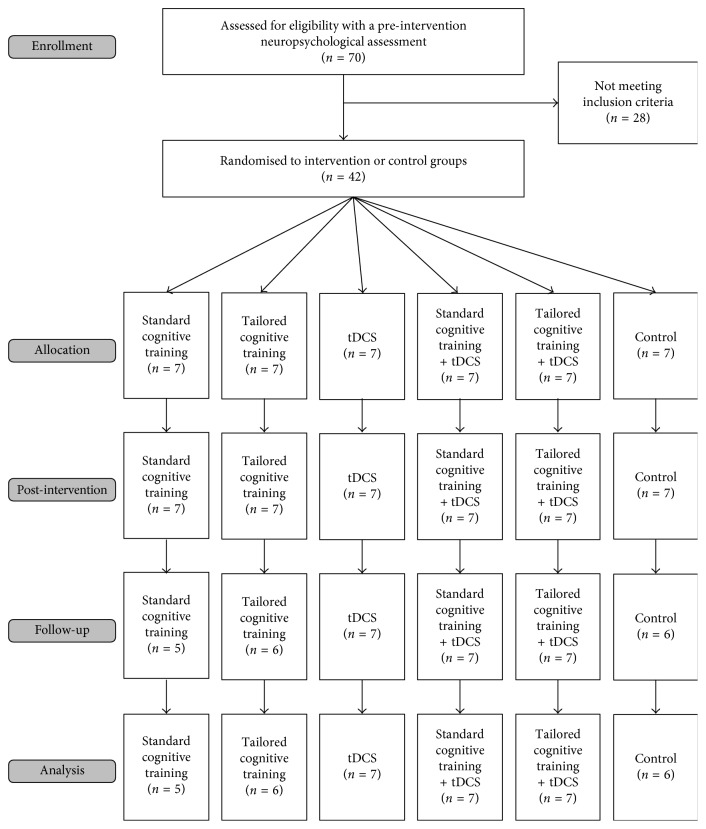
CONSORT flow diagram.

Participant recruitment and neuropsychological assessments were completed at Curtin University, Western Australia, in 2015. The following inclusion criteria applied: (1) participants diagnosed with idiopathic PD in accordance with the UK PD Brain Bank criteria, (2) presence of MCI in accordance with the Movement Disorder Society (MDS) PD-MCI Level II diagnostic criteria [13], (3) a stable response to antiparkinsonian medication at preintervention and during the course of the intervention, and (4) cognitive deficits that did not interfere with functional independence (i.e., UPDRS-II score less than 3). The following exclusion criteria applied: (1) presence of PD-Dementia, (2) recent history of brain surgery, (3) Deep Brain Stimulation (DBS) implant, (4) active skin disease on the scalp, (5) history of migraine or epilepsy, and (6) metal implants in the head/brain. 70 participants completed baseline neuropsychological assessments, with 42 meeting inclusion criteria (Figure 1). All participants completed their intervention and post-intervention neuropsychological assessments. Four participants (9.5%) did not complete follow-up assessments due to inability to travel due to disease progression (*N*=2) and lack of time (*N*=2).

### 2.3. Cognitive Training

Smartbrain Pro is an interactive computer-based training program designed to train each cognitive domain. Smartbrain Pro has been used in trials which have demonstrated improvements in cognitive functioning in Alzheimer's disease and PD [2, 14]. Smartbrain Pro was streamed directly from the Internet onto participants' home computers or onto Acer™ Aspire E3-112 portable computers via Optus™ E5251 Mini Wifi Modems (provided by the researcher). Performance was automatically monitored by the program to adjust individual difficultly levels for each activity. Participants in the standard cognitive training and standard cognitive training + tDCS groups completed a predetermined program comprising 10 activities, two activities per cognitive domain (see Supplementary Table S2). Participants in the tailored cognitive training and tailored cognitive training + tDCS groups completed activities which were individualized to their baseline neuropsychological test results. For example, a participant who demonstrated memory and executive function impairment at baseline completed only memory and executive function activities during cognitive training. The activities themselves were the same as for the standard cognitive training, and normative data were used to define each participant's degree of cognitive impairment, as described in earlier work [15].

### 2.4. Brain Stimulation

tDCS is a noninvasive brain stimulation procedure delivering low-intensity electrical currents to specific cortical areas. For participants in the tDCS, standard cognitive training + tDCS, and tailored cognitive training + tDCS groups, stimulation sessions were scheduled for the same day and time each week for 4 weeks. During each session, participants received 20 minutes of constant current 1.5 mA stimulation over left dorsal lateral prefrontal cortex (LDPFC). tDCS was delivered using the TCT™ tDCS stimulator (http://www.trans-cranial.com/) and administered with two 50 × 70 mm^2^ sponge electrodes soaked in saline solution. The anode electrode was placed over F3 according to the 10–20 international system, and the cathode electrode was placed above the left eye. Executive function and attention/working memory are most frequently impaired cognitive domains in PD [15, 16] and associated with cortical activation of the left DLPFC [5]. Previous studies demonstrate improved cognitive functioning following tDCS over left DLPFC in PD [8, 9]. Left DLPFC was therefore targeted for tDCS in this study.

### 2.5. Control Group

Participants in the control group completed baseline, post-intervention, and 12-week follow-up neuropsychological assessments, but did not complete cognitive training or tDCS.

### 2.6. Neuropsychological Assessment

Neuropsychological assessments were conducted by doctoral researchers with extensive training and experience in administration, scoring, and interpretation of neuropsychological tests in PD. The following tests were selected in accordance with MDS Task Force [13] recommendations: (1) *executive function* was assessed using the Stockings of Cambridge (SOC) subtest from CANTAB™ and the Controlled Oral Word Association Task (COWAT) [17], (2) *attention and working memory* was assessed using the Letter-Number Sequencing (LNS) [18] and the Stroop (Colour-Word Interference) Test [19], (3) *memory* was assessed using the Hopkins Verbal Learning Test-Revised (HVLT-R) immediate recall subtest [20] and the Paragraph Recall test [21], (4) *visuospatial abilities* were assessed with the Judgement of Line Orientation (JLO) test [22] and the Hooper Visual Organisation Test (HVOT) [23], and (5) language was assessed using the Boston Naming Test-Short Form (BNT) [24] and the Similarities test [18]. Global cognition was assessed using the Parkinson's Disease—Cognitive Rating Scale (PD-CRS) [25] and the Mini-Mental State Examination (MMSE) [26]. Premorbid intelligence was assessed using the Australian version of the National Adult Reading Test (AUSNART) [27]. PD-MCI was classified as less than one standard deviation (SD) below normative scores on two or more neuropsychological tests [13]. Please refer to our earlier work [15] for a detailed description of our application of the MDS Task Force criteria for classification of PD-MCI in this study's sample of participants.

Activities of daily living (ADL) and quality of life (QOL) are impacted by cognitive impairment in PD, but few nonpharmacological trials have included these outcomes. ADL and QOL were assessed by the Unified Parkinson's Disease Rating Scale (Section II) [28] and the Parkinson's Disease Questionnaire-39 (PDQ-39) [29], respectively. Depression was included as a potential covariate and assessed using the Depression, Anxiety, and Stress Scale-21 (DASS-21) [30].

### 2.7. Data Analysis

Generalized linear mixed models (GLMMs) analysed outcome variables [31] in SPSS version 22.0. Separate GLMMs were run for each outcome variable to optimise the likelihood of convergence. To control the Type 1 error rate and conserve statistical power, outcome variables were grouped by cognitive domain (e.g., executive function and memory) and a more stringent alpha level was applied (*p* < 0.025) to interaction effects. Each GLMM was assessed for statistically significant Group × Time interaction effects, main effects of Time (per group), and pairwise contrasts. Statistically significant simple main effects of Group were not of interest for this study. Significant simple main effects of Group indicate a significant difference between group outcome scores at either pre-intervention, post-intervention, or follow-up time intervals. However, this study investigated whether there was a significantly different degree of *change* (over time) on outcome variables, between groups. Therefore, pre-intervention, post-intervention, or follow-up group differences provided no statistical evidence to support the effect of interventions (or no effect of the control group) on outcome variables. Effect sizes (Hedge's *g*) were calculated using the change score method and represent a comparison between each corresponding intervention group and the control group. Sample size was determined using G^∗^Power 3. París et al. [2] and Naismith et al. [4] found moderate to large effect sizes for cognitive outcomes. To detect a moderate effect (power = 0.80 and *α* = 0.05), 54 participants were required (9 per group).

## 3. Results

No data were missing at baseline. Little's Missing Completely at Random (MCAR) test showed data missing at post-intervention (*χ*² = 23.80, *p*=0.64) and follow-up (*χ*² = 40.34, *p*=0.07) were not systematically linked to included variables. Given that GLMMs account for missing data, means and standard deviations at post-intervention and follow-up assessments were slightly adjusted by each model and do not reflect the raw data at those time points. Refer to Supplementary Tables S3, S4, and S5 for raw neuropsychological test results.

Age significantly correlated with the HVLT (*r*=−0.43, *p*=0.004), MMSE (*r*=−0.43, *p*=0.01), and PD-CRS (*r*=−0.37, *p*=0.02). Gender significantly correlated with the Stroop test (*r*=0.35, *p*=0.03). Years of education significantly correlated with Similarities (*r*=0.31, *p*=0.04) and MMSE (*r*=0.34, *p*=0.03). Premorbid IQ significantly correlated with Similarities (*r*=0.44, *p*=0.003), JLO (*r*=0.33, *p*=0.03), and MMSE (*r*=0.38, *p*=0.01). Disease duration significantly correlated with the HVOT (*r*=−0.32, *p*=0.04). LED significantly correlated with Similarities (*r*=0.33, *p*=0.03). Depression significantly correlated with Similarities (*r*=−0.39, *p*=0.01) and the PDQ-39 (*r*=0.59, *p* < 0.001). Variables with significant correlations at baseline were included as covariates in corresponding GLMMs. An analysis of variance (ANOVA) of baseline demographic statistics indicated no statistically significant differences between groups (Table 1).

A significant interaction effect was observed for SOC, indicating a differential rate of improvement in executive function between groups (*F*=3.82, *p* < 0.001). Significant improvements were identified for the standard cognitive training + tDCS group (*F*=10.73, *p* < 0.001) and tailored cognitive training + tDCS group (*F*=12.00, *p* < 0.001). No other groups improved on SOC, and no groups improved on the COWAT. Refer to Tables 2 [Table tab3]–4 for pairwise comparison statistics, effect sizes, and group baseline, post-intervention, and follow-up results.

For attention/working memory, a significant interaction effect was observed for the Stroop test (*F*=2.91, *p*=0.003). Significant improvements were identified for the tDCS group (*F*=4.06, *p*=0.02) and standard cognitive training + tDCS group (*F*=35.05, *p* < 0.001). No other groups improved on the Stroop test. A significant interaction effect was observed for LNS (*F*=4.53, *p* < 0.001). Significant improvement was identified for the tailored cognitive training group (*F*=6.62, *p*=0.002) and tailored cognitive training + tDCS group (*F*=5.11, *p*=0.01). No other groups improved on the LNS.

For memory, a significant interaction effect was observed for Paragraph Recall (*F*=2.51, *p*=0.01). Significant improvements were identified for the standard cognitive training group, (*F*=5.24, *p*=0.01), tDCS group, (*F*=17.82, *p* < 0.001), and tailored cognitive training + tDCS group (*F*=12.09, *p* < 0.001). No other groups improved on Paragraph Recall, and no groups improved on HVLT.

For language, a significant interaction effect was observed for the Similarities test (*F*=3.25, *p*=0.001). Significant improvements were identified for the standard cognitive training + tDCS group (*F*=5.23, *p*=0.01) and tailored cognitive training + tDCS group (*F*=17.43, *p* < 0.001). No other groups improved on the Similarities test, and no groups improved on the BNT.

For visuospatial abilities, a significant interaction effect was observed for JLO (*F*=3.76, *p* < 0.001). However, a significant decline was identified for the standard cognitive training group (*F*=6.57, *p*=0.002). Therefore, no groups improved on JLO, and no groups improved on HVOT.

No groups improved on measures of global cognition (MMSE and PD-CRS).

For QOL, a significant interaction effect was observed for the PDQ-39 (*F*=2.96, *p*=0.003). Significant improvements were identified for the standard cognitive training group (*F*=7.21, *p*=0.001) and tailored cognitive training group (*F*=12.48, *p* < 0.001). No other groups improved on QOL.

For ADL, a significant interaction effect was observed for the UPDRS-II (*F*=1.96, *p*=0.04). Significant improvements were identified for the standard cognitive training group (*F*=11.29, *p* < 0.001) and standard cognitive training + tDCS group (*F*=3.40, *p*=0.04). No other groups improved on ADL.

## 4. Discussion

In support of the therapeutic potential of cognitive training and tDCS, differential rates of improvements in cognition, ADL, and QOL were observed across intervention groups. The control group did not improve on any outcome measures.

The standard cognitive training group improved on memory, ADL, and QOL. Previous standard cognitive training studies report improved memory [2] and ADL in PD [32], but this study is the first to report improvement in QOL. París et al. [2] used the same computer-based cognitive training program (Smartbrain Pro) and the same QOL outcome measure (PDQ-39), but their participants did not improve. This may reflect a ceiling effect as half the participants in París et al.'s [2] cognitive training group were identified as having normal cognition. Nonetheless, ADL and QOL are frequently impaired in PD and associated with cognitive decline [33, 34]. The current findings indicate that standard cognitive training improves ADL and QOL for those with PD.

The tailored cognitive training group improved on attention/working memory and QOL. One tailored cognitive training study has reported “attentional improvements,” evidenced by increased neural resting state activity (measured by fMRI) in the superior parietal and prefrontal dorsolateral cortices following training [5]. The current study is the first to report improvements in QOL following tailored cognitive training in participants with PD or PD-MCI. Despite limited evidence in PD, a Cochrane review of cognitive training for people with mild to moderate dementia reported positive effects of cognitive training for QOL (and cognitive function) [35]. The positive results in dementia and in the current study indicate that future studies should explore the potential of tailored cognitive training to improve QOL in PD-MCI.

The tDCS group improved on attention/working memory and memory. Recent studies report significant improvements in attention/working memory in PD [8] and attentional/executive abilities [9, 10]. The current study is the first to demonstrate memory improvement following tDCS in PD-MCI. In accordance with the “dual syndrome hypothesis” [36], if participants in the current study had the APOE allelic genetic abnormality associated with memory deficits in the posterior cortex, the Scaffolding Theory of Ageing and Cognition [37] suggests that their impaired posterior cortical function may have led to compensatory activation of the prefrontal cortices (i.e., left DLPFC) to account for increased cognitive demand during complex tasks (i.e., neuropsychological assessments). Anodal tDCS may have therefore enhanced compensatory activation of the left DLPFC, leading to increased neural activity of frontal functions that were associated with improved memory performance in PD-MCI.

The standard cognitive training + tDCS group improved on executive function, attention/working memory, and ADL. Multiple uncontrolled studies combined standard cognitive training with tDCS, but the results vary. Biundo et al. [11] reported a decline in executive skills and improved attention and memory. Conversely, research in Alzheimer's disease paired repetitive transcranial magnetic stimulation (rTMS) with standard cognitive training and reported improved global cognition [38]. However, different methods of noninvasive stimulation, both anodal tDCS and high-frequency rTMS, increase cortical excitability to improve cognitive functioning [7]. In accordance with Mowszowski et al. [39], combining standard cognitive training with tDCS in the current study may have resulted in “positive plasticity” to alleviate cognitive deficits. Standard cognitive training may have stimulated and strengthened existing neural connections (synaptogenesis), while tDCS provided compensatory activation of a cortical region (left DLPFC) associated with higher-order cognition and functional improvement in ADL.

This is the first standard cognitive training and tDCS study to report language improvements in PD. Improved language abilities may be explained by the overlap between the language skills needed to complete the Similarities outcome test and those needed to complete the cognitive training program. During the language activities, participants finished sentences by selecting an appropriate word and determining the relationship between a group of words by applying a semantic category to those words. Successful completion of the Similarities test also involves application of semantic word categories to describe the most appropriate relationship between a set of words [18]. Participants in the standard cognitive training + tDCS group may have therefore trained and improved language skills that were most beneficial for successful performance on the Similarities language test. There is mounting evidence indicating that some people with PD demonstrate language impairment [16, 40], and the current study suggests that combining standard cognitive training with tDCS may alleviate this deficit.

The tailored cognitive training + tDCS group improved on executive function, attention/working memory, and memory. Among studies that have examined these interventions independently, several reports improved executive function and attention/working memory in PD [5, 9]. The current study is the first to report memory improvements following tailored cognitive training and tDCS in PD. Memory impairment is common in PD and may predict progression to PD-Dementia [41]. Future clinical trials of tDCS and tailored cognitive training need to include standardised memory outcomes and interventions targeting memory impairment in PD.

The current study is also the first to report improved language abilities following tailored cognitive training and tDCS in PD. For the tailored cognitive training + tDCS group, language improvements were observed on the Similarities test, but not the BNT. The MDS Task Force classifies the Similarities test as a measure of language abilities [13]. However, the Similarities test is a subtest of the verbal IQ index of the WAIS battery and involves abstract reasoning [18]. Abstract reasoning is a higher-order cognitive ability associated with executive function and involves ordering, comparing, analysing, and synthesizing information [42]. When completing the Similarities test, participants need to describe in what ways are two concepts/words alike, which requires the use of abstract reasoning (an executive skill) to synthesise information related to both concepts/words. As a task requiring executive function, completing the Similarities test may involve increased activation of left DLPFC, which was also the target of tDCS for this group. Participants in this group also demonstrated impaired executive function (lowest baseline SOC score) and completed cognitive training tasks tailored to executive function skills. Pairing this form of tailored cognitive training with tDCS applied to left DLPFC may have increased cortical activity associated with improved performance on SOC *and* Similarities, tasks involving executive *and* language abilities. According to the theoretical model proposed by Kim and Kim [43], combining a stimulation and compensation-focussed intervention (tailored cognitive training) with another compensation-focussed intervention (tDCS) may have provided optimal conditions for neuronal plasticity, which led to improved performance across several cognitive domains.

There are limitations to the current study. Several outcomes did not improve across intervention groups, which may be due to a number of reasons. Despite selecting outcomes in accordance with MDS Task Force recommendations [13], a lack of sensitivity of some cognitive tests for detecting change in PD may have contributed to nonsignificant improvement for those tests (e.g., HVLT, BNT, and MMSE). [42] Researchers should consult compendiums of neuropsychological tests [42] to ensure that sensitive outcomes are included in future clinical trials. The cognitive training and tDCS parameters used in this study may have also impacted nonsignificant results. No improvements were observed for visuospatial abilities as measured by HVOT and JLO. These tests involve perceptual organisation (HVOT) and estimation of angled lines (JLO), but the visuospatial activities in the cognitive training interventions involved different visuospatial skills (e.g., identifying coordinates and time ranges on an analog clock). Furthermore, the tDCS in this study stimulated a cortical region (left DLPFC) that is not associated with visuospatial performance. Several studies report more dominant involvement of the right posterior hemisphere during completion of HVOT and JLO [44, 45]. It is therefore likely that the cognitive training tasks and site of tDCS were not conducive to improved visuospatial abilities. It is also important to note that two participants in the standard cognitive training group with high JLO scores at pre-intervention dropped out of the study preceding the follow-up assessment, which may account for this group's significant decline in JLO performance at follow-up. This study was also somewhat underpowered, which may have impacted nonsignificant outcome effects. Lastly, exposure was not matched between intervention groups. Participants allocated to the cognitive training groups (standard or tailored) completed 12 sessions of training. Whereas, participants in the cognitive training + tDCS groups completed 12 sessions of cognitive training *and* 4 sessions of tDCS. Completing both interventions exposed participants to a greater number of therapeutic sessions designed to improve cognition, which may have produced additive beneficial effects on neuropsychological outcomes. Future studies should account for these methodological parameters when exploring the therapeutic potential of cognitive training and tDCS in PD and PD-MCI.

## 5. Conclusions

This study provides evidence in support of cognitive training, tDCS, and cognitive training combined with tDCS for PD-MCI. The rate of participant attrition was low (<10%), and cognitive performance was measured in line with MDS Task Force recommendations for Level II diagnostic criteria of PD-MCI [13]. Overall, a greater number of outcomes improved for the groups that received standard or tailored cognitive training combined with tDCS. These findings suggest that cognitive training combined with tDCS may provide optimal conditions for neuronal plasticity, leading to improvements in cognition and functional outcomes for those with PD-MCI.

## Figures and Tables

**Table 1:** Baseline demographic information for the intervention and control groups.

	Standard CT		Tailored CT		tDCS		Standard CT + tDCS		Standard CT + tDCS		Control		Group differences	
Outcome	*M*	SD	*M*	SD	*M*	SD	*M*	SD	*M*	SD	*M*	SD	*F*	*p*
Gender (%, ♀)	43% (*N*=3)		57% (*N*=4)		71% (*N*=5)		71% (*N*=5)		71% (*N*=5)		57% (*N*=4)		0.36	0.87
Age^++^	68.14	8.69	65.57	5.20	72	6.45	63.57	15.68	67.43	6.37	72.29	6.21	0.80	0.56
Education^++^	13.57	2.64	12.21	2.83	13.57	3.69	15.50	3.35	15.86	1.35	11.71	2.98	2.34	0.06
Premorbid IQ	103.29	6.96	107.21	12	108.21	5.83	111.96	4.37	111.08	3.59	103.64	7.53	1.76	0.15
Disease Duration^++^	5.29	4.23	5.79	4.97	5.50	5.66	6.79	4.60	4.43	2.70	5.36	4.14	0.20	0.96
LED	295	313.40	383	178.62	573.29	586.25	350.71	322.37	464.29	358.78	292.88	274.51	0.64	0.68
DASS-D	2.29	2.56	1.29	1.50	3	2	3	5.07	3.29	4.11	2.71	3.15	0.34	0.89

*M* = mean; SD = standard deviation; *F* = ANOVA; *p* = level of statistical significance; *N* = number of participants; LED = levodopa equivalent dose; DASS-D = Depression, Anxiety, and Stress Scale (Depression subscale); IQ = intelligence quotient; CT = cognitive training; tDCS = transcranial direct current stimulation; ♀ = male gender; ++ = years.

**Table 2:** Standard cognitive training and tailored cognitive training group cognitive performance at baseline, post-intervention, and follow-up.

	Standard cognitive training						Pairwise comparison statistics					
	Baseline		Post-intervention		Follow-up		Baseline to post-intervention			Baseline to follow-up		
Outcome	*M*	SD	*M*	SD	*M*	SD	*t*	*p*	*g* ^a^	*t*	*p*	*g* ^a^
COWAT	38.86	13.89	44.14	11.40	42.20	13.60	1.81	0.07	0.70	1.31	0.19	−0.16
SOC	8.17	0.64	6.43	2.52	7.69	2.33	−1.90	0.06	−1.19	−0.51	0.61	−0.70
LNS	18.73	2.54	19.02	4.77	17.14	3.97	0.24	0.81	−0.04	−1.26	0.21	−0.42
Stroop test	33.83	6.20	36.11	6.76	36.16	5.31	0.82	0.42	0.35	1.67	0.10	−0.31
HVLT	22.08	5.41	26.94	5.35	27.44	2.33	3	0.003^b^	0.46	2.76	0.01^b^	0.44
Paragraph Recall	5	2.20	6.36	2.28	7.09	2.71	1.36	0.18	0.62	3.16	**0.002** ^∗^	0.93
BNT	14.14	1.72	13.86	0.98	13.60	1.17	−0.73	0.43	−0.55	−0.79	0.43	0.01
Similarities	22.14	3.05	23.71	3.31	21.89	1.17	1.51	0.25	0.28	−0.30	0.76	−0.70
JLO	25.61	3.39	24.90	4.35	20.61	3.42	−0.71	0.48	−0.08	−2.94	**0.004** ^∗^	−1.19
HVOT	24.49	3.76	25.21	3.45	26.07	2.96	0.84	0.40	0.06	1.38	0.17	0.36
MMSE	26.95	2.09	26.80	1.78	28.19	2.24	−0.19	0.85	0.41	0.86	0.39	−0.09
PD-CRS	89.42	10.28	96.13	9.12	100.09	8.94	1.42	0.16	0.44	2.29	0.02^b^	0.28
PDQ-39	24.20	10.07	20.68	10.76	25.60	9.95	−3.05	**0.003** ^∗^	0.24	0.71	0.48	0.06
UPDRS-II	0.96	0.77	0.73	0.74	0.85	0.74	−4.60	**0.001** ^∗∗^	0.33	−1.55	0.13	0.36

	Tailored cognitive training						Pairwise comparison statistics					
	Baseline		Post-intervention		Follow-up		Baseline to post-intervention			Baseline to follow-up		

Outcome	*M*	SD	*M*	SD	*M*	SD	*t*	*p*	*g* ^a^	*t*	*p*	*g* ^a^
COWAT	32.43	16.59	34.71	12.19	37.17	21.27	0.56	0.58	0.42	0.96	0.34	−0.07
SOC	6.43	2.33	6.55	2.07	5.26	2.84	0.14	0.89	−0.51	−0.98	0.33	−0.85
LNS	17.39	4.43	18.87	3.10	19.81	2.70	1.95	0.06	0.19	3.30	**0.001** ^∗^	0.30
Stroop test	31.89	9.01	35.17	10.65	31.83	9.49	1.42	0.16	0.34	−0.03	0.98	−0.51
HVLT	22.15	5.35	24.58	6.15	26.52	6.36	2.41	0.02^b^	0.05	2.93	0.004^b^	0.27
Paragraph Recall	5.64	2.20	7.36	3.50	7.23	3.21	2.21	0.03^b^	0.58	1.35	0.18	0.74
BNT	13.14	1.25	13.86	1.25	13.78	1.05	1.48	0.14	0.09	1.14	0.26	0.66
Similarities	22.11	2.47	23.71	3.31	22.72	1.52	−0.03	0.98	−0.36	0.51	0.61	−0.54
JLO	20.25	3.55	22.97	4.32	22.32	3.87	2.13	0.04^b^	0.49	1.94	0.06	−0.06
HVOT	20.37	3.76	23.22	3.45	23.19	5.35	3.35	0.001^b^	0.61	2.62	0.01^b^	0.65
MMSE	25.49	2.44	26.92	2.54	25.85	1.20	−2.70	0.01^b^	0.98	0.55	0.59	−0.45
PD-CRS	86.58	12.72	95.29	21.44	98.61	18.28	2.31	0.02^b^	0.39	3.64	0.001^b^	0.35
PDQ-39	21.41	7.39	18.09	5.19	18.21	8.65	−2.46	**0.02** ^∗^	0.26	−2.44	**0.02** ^∗^	0.30
UPDRS-II	0.68	0.32	0.80	0.40	0.71	0.27	0.88	0.38	−0.06	0.17	0.87	−0.24

*M* = mean; SD = standard deviation; *t* = t statistic; a = effect size computed using change scores with the control group, positive effect favours corresponding intervention group; b = not statistically significant due to nonsignificant main effect; ^∗^ = *p* < 0.05; ^∗∗^ = *p* < 0.001; tDCS = transcranial direct current stimulation; COWAT = Controlled Oral Word Association Test; SOC = Stockings of Cambridge; LNS = Letter-Number Sequencing; HVLT = Hopkin's Verbal Learning Test; BNT = Boston Naming Test; JLO = Judgement of Line Orientation; HVOT = Hooper's Visual Orientation Test; MMSE = Mini-Mental State Examination; PD-CRS = Parkinson's Disease—Cognitive Rating Scale; UPDRS-II = Unified Parkinson's Disease Rating Scale—Section II (ADL); PDQ-39 = Parkinson's Disease Questionnaire-39.

**Table 3 tab3:** tDCS and standard cognitive training + tDCS group cognitive performance at baseline, pos-tintervention, and follow-up.

	tDCS						Pairwise comparison statistics					
	Baseline		Post-intervention		Follow-up		Baseline to post-intervention			Baseline to follow-up		
Outcome	*M*	SD	*M*	SD	*M*	SD	*t*	*p*	*g* ^a^	*t*	*p*	*g* ^a^
COWAT	30.86	16.14	36	15.11	30.57	17.57	1.86	0.06	0.58	−0.17	0.86	−0.30
SOC	5.50	1.35	6.50	1.62	7	1.88	2.12	0.04^b^	−0.13	1.94	0.06	0.14
LNS	14.82	5.99	16.11	6.39	16.18	4.74	1.70	0.09	0.12	1.15	0.25	0.13
Stroop test	21.38	6.76	27.66	10.41	26.52	7.47	2.09	**0.04** ^∗^	0.65	2.41	**0.02** ^∗^	0.01
HVLT	20.33	8.27	25.33	6.28	23.33	7.45	4.45	0.001^b^	0.45	3.71	0.001^b^	0.03
Paragraph Recall	4	2.23	6.29	2.05	4.36	1.72	4.73	**0.001** ^∗∗^	1.11	0.70	0.49	0.28
BNT	12.57	1.06	13.71	1.83	13.29	1.67	2.23	0.03^b^	0.30	1.63	0.11	0.65
Similarities	22.37	2.68	23.42	2.41	21.38	1.67	1.29	0.20	0.13	−0.76	0.45	−1.25
JLO	21.30	7.90	22.44	5.51	24.59	4.80	0.80	0.43	0.24	2.31	0.02^b^	0.18
HVOT	20.99	3.76	22.42	3.45	20.99	3.26	1.68	0.10	0.24	0.00	1	−0.01
MMSE	24.31	1.54	26.02	1.64	25.45	2.43	2.29	0.02^b^	1.36	1.84	0.07	0.55
PD-CRS	74.76	17.57	85.04	18.10	79.04	20.59	5.10	0.001^b^	0.53	1.51	0.14	−0.06
PDQ-39	23.04	8.45	18.09	5.19	18.21	8.65	−0.96	0.34	0.22	−1.11	0.27	0.37
UPDRS-II	1.27	0.56	1.06	0.66	1.23	0.66	−2.25	0.03^b^	0.32	−0.48	0.63	0.24

	Standard cognitive training + tDCS						Pairwise comparison statistics					
	Baseline		Post-intervention		Follow-up		Baseline to post-intervention			Baseline to follow-up		

Outcome	*M*	SD	*M*	SD	*M*	SD	*t*	*p*	*g* ^a^	*t*	*p*	*g* ^a^
COWAT	37.71	9.88	46.14	4.85	39.86	11.26	3.23	0.002^b^	1.33	0.44	0.66	−0.22
SOC	7.43	1.41	9.57	1.64	9.14	2.09	4.62	**0.001** ^∗∗^	0.41	2.29	**0.02** ^∗^	0.23
LNS	18.54	2.17	18.87	2.47	18.56	2.60	0.34	0.73	−0.04	0.01	0.99	−0.14
Stroop test	24.52	9.30	30.52	8.29	33.81	9.01	2.23	**0.03** ^∗^	0.60	5.76	**0.001** ^∗∗^	0.24
HVLT	26.51	4.08	28.51	5.10	29.94	4.85	1.43	0.16	−0.03	2.59	0.01^b^	0.09
Paragraph Recall	6	2.07	8.21	1.38	6.43	2.23	2.30	0.02^b^	1.29	3.25	0.002^b^	0.57
BNT	13.29	1.59	14.43	0.74	14	0.93	1.75	0.08	0.39	1.83	0.07	0.77
Similarities	21.59	2.49	23.51	1.72	21.02	2.31	2.70	**0.01** ^∗^	0.59	2.02	0.06	−0.95
JLO	22.83	5.43	24.55	4.74	22.98	7	1.58	0.12	0.37	0.11	0.91	−0.23
HVOT	23.82	3.79	25.11	3.45	24.11	3.29	1.51	0.14	0.21	1.03	0.78	0.05
MMSE	27.02	1.72	26.87	1.30	27.73	1.33	−0.20	0.84	0.46	1.36	0.18	0.50
PD-CRS	87.08	16.93	98.79	13.03	94.94	18.02	5.30	0.001^b^	0.71	2.69	0.01^b^	0.10
PDQ-39	20.05	11.74	16.33	7.84	19.48	10.87	−1.82	0.07	0.27	−0.30	0.77	−0.09
UPDRS-II	1	0.48	0.62	0.53	0.77	0.32	−2.51	**0.01** ^∗^	0.55	−1.74	0.08	0.51

*M* = mean; SD = standard deviation; *t* = t statistic; a = effect size computed using change scores with the control group, positive effect favours corresponding intervention group; b = not statistically significant due to nonsignificant main effect; ^∗^ = *p* < 0.05; ^∗∗^ = *p* < 0.001; tDCS = transcranial direct current stimulation; COWAT = Controlled Oral Word Association Test; SOC = Stockings of Cambridge; LNS = Letter-Number Sequencing; HVLT = Hopkin's Verbal Learning Test; BNT = Boston Naming Test; JLO = Judgement of Line Orientation; HVOT = Hooper's Visual Orientation Test; MMSE = Mini-Mental State Examination; PD-CRS = Parkinson's Disease—Cognitive Rating Scale; UPDRS-II = Unified Parkinson's Disease Rating Scale—section II (ADL); PDQ-39 = Parkinson's Disease Questionnaire-39.

**Table 4:** Tailored cognitive training + tDCS and control group cognitive performance at baseline, post-intervention, and follow-up.

	Tailored cognitive training + tDCS						Pairwise comparison statistics					
	Baseline		Post-intervention		Follow-up		Baseline to post-intervention			Baseline to follow-up		
Outcome	*M*	SD	*M*	SD	*M*	SD	*t*	*p*	*g* ^a^	*t*	*p*	*g* ^a^
COWAT	32.14	9.22	36.29	8.45	35.86	11.50	1.74	0.08	0.70	1.77	0.08	−0.12
SOC	5.29	1.03	7	2.07	8.57	2.20	2.29	**0.02** ^∗^	0.19	3.86	**0.001** ^∗∗^	0.92
LNS	17.63	2.54	18.25	1.99	19.24	1.96	0.72	0.47	0.02	2.21	**0.03** ^∗^	0.22
Stroop test	29.95	11.50	30.66	6.97	32.38	7.10	0.18	0.86	0.14	0.87	0.39	−0.17
HVLT	21.04	2.70	24.90	2.28	25.19	5.30	5.21	0.001^b^	0.37	2.78	0.01^b^	0.22
Paragraph Recall	3.21	1.43	5.71	1.64	6.43	2.23	4.60	**0.001** ^∗∗^	1.36	3.25	**0.002** ^∗^	1.75
BNT	14	1.43	14.29	1.03	14.57	0.50	0.86	0.39	−0.18	−0.42	0.68	0.73
Similarities	22.89	3.18	26.03	2.25	20.75	2.20	3.73	**0.001** ^∗∗^	1.06	−2.15	**0.03** ^∗^	−1.58
JLO	22.33	7.71	22.47	6.31	22.04	7.13	0.12	0.91	0.06	−0.55	0.59	−0.28
HVOT	21.51	3.79	24.08	3.71	24.37	3.29	3.02	0.003^b^	0.54	2.76	0.01^b^	0.62
MMSE	26.37	1.99	26.51	1.38	26.37	1.54	0.21	0.84	0.61	0.00	1	0.19
PD-CRS	85.56	12.91	93.84	11.69	90.99	8.98	2.41	0.02^b^	0.51	2.66	0.01^b^	−0.02
PDQ-39	25.62	14.63	27.25	10.52	20.44	11.32	0.53	0.60	−0.14	−1.64	0.11	0.27
UPDRS-II	1.17	0.56	0.97	0.48	1.16	0.48	−1.47	0.14	0.34	−0.01	0.99	0.22

	Control						Pairwise comparison statistics					
	Baseline		Post-intervention		Follow-up		Baseline to post-intervention			Baseline to follow-up		

Outcome	*M*	SD	*M*	SD	*M*	SD	*t*	*p*	*g* ^a^	*t*	*p*	*g* ^a^
COWAT	30.14	15.45	27.43	9.94	36	19.16	−0.87	0.39	—	1.81	0.07	—
SOC	6	2.84	7.28	2.27	7.21	1.84	1.89	0.06	—	1.75	0.08	—
LNS	14.30	6.07	14.82	5.78	14.97	5.02	0.77	0.44	—	1.04	0.30	—
Stroop test	19.03	10.07	18.60	9.38	24.24	20.56	−0.34	0.74	—	0.86	0.39	—
HVLT	21.26	5.51	23.40	5.75	24.04	5.71	1.49	0.14	—	2.84	0.01^b^	—
Paragraph Recall	4.07	2.41	4.07	1.80	3.81	1.30	0.00	1	—	−0.36	0.72	—
BNT	12.43	1.99	13	1.76	11.87	1.84	3.06	0.003^b^	—	−0.87	0.39	—
Similarities	18.80	1.99	19.55	1.96	20.70	2.43	1.06	0.29	—	1.85	0.07	—
JLO	21.10	6.92	20.82	7.55	23.56	8.18	−0.37	0.71	—	1.38	0.17	—
HVOT	23.60	4.06	24.10	3.71	23.66	3.38	0.54	0.59	—	0.05	0.96	—
MMSE	24.38	2.07	24.38	2.16	25.82	2.31	−1.37	0.17	—	0.71	0.48	—
PD-CRS	76.50	19.35	77.50	14.81	82.22	20.21	0.33	0.74	—	3.21	0.002^b^	—
PDQ-39	24.02	14.52	23.76	14.92	24.59	11.81	−0.35	0.73	—	0.39	0.70	—
UPDRS-II	1.18	0.69	1.25	0.94	1.35	0.93	0.71	0.48	—	1.04	0.30	—

*M* = mean; SD = standard deviation; *t* = t statistic; a = effect size computed using change scores with the control group, positive effect favours corresponding intervention group; b = not statistically significant due to nonsignificant main effect; ^∗^ = *p* < 0.05; ^∗∗^ = *p* < 0.001; tDCS = transcranial direct current stimulation; COWAT = Controlled Oral Word Association Test; SOC = Stockings of Cambridge; LNS = Letter-Number Sequencing; HVLT = Hopkin's Verbal Learning Test; BNT = Boston Naming Test; JLO = Judgement of Line Orientation; HVOT = Hooper's Visual Orientation Test; MMSE = Mini-Mental State Examination; PD-CRS = Parkinson's Disease—Cognitive Rating Scale; UPDRS-II = Unified Parkinson's Disease Rating Scale—section II (ADL); PDQ-39 = Parkinson's Disease Questionnaire-39.

## Supplementary Materials

Supplementary Materials Five tables are included in supplementary material. The CONSORT checklist is reported in Table S1. A summary of the cognitive training activities is reported in Table S2. Baseline, post-intervention, and follow-up neuropsychological test results are reported in Tables S3, S4, and S5, respectively.
